# DNA Damage, Cell Cycle Arrest, and Apoptosis Induction Caused by Lead in Human Leukemia Cells

**DOI:** 10.3390/ijerph13010056

**Published:** 2015-12-22

**Authors:** Clement G. Yedjou, Hervey M. Tchounwou, Paul B. Tchounwou

**Affiliations:** Natural Chemotherapeutics Research Laboratory, NIH-Center for Environmental Health, College of Science, Engineering and Technology, Jackson State University, 1400 Lynch Street, P.O. Box 18540, Jackson, MS 39217, USA; hervey.tchounwou@students.jsums.edu (H.M.T.); paul.b.tchounwou@jsums.edu (P.B.T.)

**Keywords:** lead nitrate, HL-60 cells, DNA damage, apoptosis, cell cycle, cellometer vision

## Abstract

In recent years, the industrial use of lead has been significantly reduced from paints and ceramic products, caulking, and pipe solder. Despite this progress, lead exposure continues to be a significant public health concern. The main goal of this research was to determine the *in vitro* mechanisms of lead nitrate [Pb(NO_3_)_2_] to induce DNA damage, apoptosis, and cell cycle arrest in human leukemia (HL-60) cells. To reach our goal, HL-60 cells were treated with different concentrations of Pb(NO_3_)_2_ for 24 h. Live cells and necrotic death cells were measured by the propidium idiode (PI) assay using the cellometer vision. Cell apoptosis was measured by the flow cytometry and DNA laddering. Cell cycle analysis was evaluated by the flow cytometry. The result of the PI demonstrated a significant (*p* < 0.05) increase of necrotic cell death in Pb(NO_3_)_2_-treated cells, indicative of membrane rupture by Pb(NO_3_)_2_ compared to the control. Data generated from the comet assay indicated a concentration-dependent increase in DNA damage, showing a significant increase (*p* < 0.05) in comet tail-length and percentages of DNA cleavage. Data generated from the flow cytometry assessment indicated that Pb(NO_3_)_2_ exposure significantly (*p* < 0.05) increased the proportion of caspase-3 positive cells (apoptotic cells) compared to the control. The flow cytometry assessment also indicated Pb(NO_3_)_2_ exposure caused cell cycle arrest at the G_0_/G_1_ checkpoint. The result of DNA laddering assay showed presence of DNA smear in the agarose gel with little presence of DNA fragments in the treated cells compared to the control. In summary, Pb(NO_3_)_2_ inhibits HL-60 cells proliferation by not only inducing DNA damage and cell cycle arrest at the G_0_/G_1_ checkpoint but also triggering the apoptosis through caspase-3 activation and nucleosomal DNA fragmentation accompanied by secondary necrosis. We believe that our study provides a new insight into the mechanisms of Pb(NO_3_)_2_ exposure and its associated adverse health effects.

## 1. Introduction

Lead is a malleable substance found naturally in the Earth’s crust, and also extracted from other metals, such as ore, copper, and silver. Throughout history, lead has been used in various industrial applications including the manufacturing of fossil fuels, paint, plumbing materials, batteries, and cosmetics [1]. Despite its beneficial industrial uses, lead has caused environmental contamination of the air, water, and soil [1]. Environmental contamination of lead is often toxic to both human and animal health. Recent studies conducted by US Environmental Protection Agency reported that children and pregnant women are the population most vulnerable to the toxic effects of lead exposure [1]. A widely cited scientific paper suggests that lead exposure during pregnancy can inversely affect fetal growth, neurological development, and cause spontaneous abortion [2]. Worldwide, lead is considered as a serious occupational hazard [3]. Previous study in our laboratory showed that treatment of human leukemia (HL-60) cells with lead nitrate significantly increased lipid hydroperoxide levels, a major degradation product of unsaturated phospholipids and glycolipids [4]. Consistent with our previous finding, lead is reported to cause oxidative stress by generating the release of reactive oxygen species (ROS), such as superoxide radicals, hydrogen peroxide and hydroxyl radicals and lipid peroxides [5,6]. It has been shown that exposure to lead enhances intracellular ROS production, lipid peroxidation, and tissue damage in animal reproductive systems [7]. It has also been reported that lead exposure alters the activities of antioxidant enzymes, such as glutathione peroxidase, catalase and superoxide dismutase in various experimental animals [8,9]. Bressler and collaborators reported that lead exposure causes neurotoxic effects, such as behavioral abnormalities, learning impairment, decreased hearing, and impaired cognitive functions in human and experimental animals [10]. Maternal lead exposure has been linked to a large number of adverse health effects of developing fetus [2] and associated with increases in maternal blood pressure in pregnant women [11].

Although much more research is needed to determine the minimum levels of exposure that cause negative health consequences, scientific data have provided enough information to conclude that lead poses a health risk to both pregnant women and their babies [12]. Scientific data also highlight oxidative stress as a key factor in lead-associated kidney damage, but it has been unclear how the stress is generated [13]. Lead affects numerous organ systems in the body, but its specific mechanisms of damage are not always known. Thus far, many scientific reports on lead have been focused on its immunotoxic effects in animal experiments and people with occupational exposure to lead [14,15]. Presently, there are limited reports on the influence of lead exposure on lymphocytes. Therefore, the present study was designed to evaluate the *in vitro* mechanisms of lead induces toxicity, DNA damage, cell cycle arrest, and apoptosis of human leukemia (HL-60) cells.

## 2. Materials and Methods

### 2.1. Chemicals and Media

We obtained reference solution (1000 ± 10 ppm) of lead nitrate [Pb(NO_3_)_2_] (CAS No. 10099-74-8, Lot No. 981735-24) with a purity of 100% from Fisher Scientific in Fair Lawn, New Jersey. Growth medium RMPI 1640 containing 1 mmol/L l-glutamine was purchased from Gibco BRL products (Grand Island, NY, USA). Fetal bovine serum (FBS), phosphate buffered saline (PBS), and propidium assay were obtained from Sigma Chemical Company (St. Louis, MO, USA). Active caspase-3 kit was obtained from BD Biosciences (Pharmingen, CA, USA).

### 2.2. Cell/Tissue Culture

The HL-60 cell line was originally derived from a 36 year-old Caucasian female with acute promyelocytic leukemia (APL). In the laboratory, HL-60 cells were maintained as previously described [16]. Briefly, cells were grown in RMPI 1640 medium containing 1 mmol/L l-glutamine (GIBCO/BRL, Gaithersburg, MD, USA) and supplemented with 10% (*v*/*v*) fetal bovine serum (FBS), and 1% (w/v) penicillin/streptomycin. Cells were observed under the inverted microscope daily following by incubation in a humidified 5% CO_2_ incubator at 37 °C. Three times a week, cells were manually counted using a hemocytometer and diluted to maintain a density of 5 × 10^5^ cells/mL.

### 2.3. Biochemical Test for Live Cells and Necrotic Death Cells by Cellometer Imaging

To determine the cytotoxic effect of Pb(NO_3_)_2_ *in vitro*, we measured cell viability by propidium iodine (PI) staining using the Cellometer Imaging system. Briefly, 1 × 10^6^ cells/mL per treatment in 3 replicates was harvested, washed, and re-suspended in 1 mL of culture media. Five microliters of propidium iodide (PI) was added to 100 μL of cell suspension taken out from each sample. Samples were gently mixed and incubated for 20 min at room temperature in dark. Samples were mixed again and 20 μL of each sample was loaded into the cellometer counting chamber. Samples in the cellometer counting chamber were analyzed by the Cellometer Vision. Both cell concentration and viability were determined with the Vision software. Hence, the Cellometer Vision-based imaging was successfully used in our laboratory to identify live and necrotic death cells [17].

### 2.4. Biochemical Test for DNA Damage by Comet Assay

The comet assay was carried out by the method previously described by Collins and his collaborators [18,19] with some modifications [20]. Briefly, 1 × 10^5^ cells were seeded and allowed to attach for at least 24 h before treatment with lead nitrate. At the end of treatment, cells were centrifuged, washed with PBS free calcium and magnesium, and re-suspended in 100 µL PBS. Five hundred microliters of melted LMAgarose plus 50 µL of the cells suspension were mixed and 75 µL was pipetted onto a pre-warmed cometslide. The cometslides were placed flat in the dark at 4 °C for 10 min and then immersed in prechilled lysis solution at 4 °C for 40 min. After the lysis, the slides were immersed in Alkaline Solution for 40 min at room temperature in the dark. Next, the samples were electrophoresed at low voltage (300 mA, 25 V, 4 °C) for 20 min and stained with SYBR Green designed for 6 h. Samples were viewed with an Olympus fluorescence microscope and analyzed using LAI’s Comet assay Analysis System software (Loates Associates, Inc. Westminster, MD, USA).

### 2.5. Biochemical Test for Cell Cycle Distribution by Flow Cytometry

For this experiment, cells were cultured in 6 well plates. Control (untreated) and treated cells with Pb(NO_3_)_2_ were harvested from 6 well plates after 24 h of exposure. Harvested cells were washed twice with phosphate-buffered saline (PBS) and 5 × 10^6^ cells/mL per sample were fixed with 70% methanol on ice for 10 min. After cells fixation, cell pellet was then suspended in 0.5 mL PBS containing propidium iodide (50 μg/mL) and DNase-free RNase (100 μg/mL) for 15 min. The relative number of cells in the different phases was assessed by flow cytometry (FACS Calibar; Becton-Dickinson) using CellQuest software, and the percentages of cells calculated in G0/G1, S and G2/M phases of the cell cycle.

### 2.6. Biochemical Test for Apoptosis by DNA Laddering Assay

We recently demonstrated lead nitrate induced apoptosis in human leukemia (HL-60) cells through induction of phosphatidylserine externalization and caspase-3 activation [16]. To further confirm that lead nitrate is a potential apoptotic agent, we performed DNA Laddering Assay. Briefly, 2 mL of cells (1 × 10^6^ cells/mL) untreated or treated with 10, 20 and 30 µg/mL of lead nitrate were incubated for 24 h. After the incubation period, cellular DNA was extracted using genomic DNA isolation reagents from Roche Molecular Biochemicals (Indianapolis, IN) according to the manufacturer’s protocol. After extraction, samples were placed into the well of agarose gel (1.2%, w/v) and electrophoresed at 75 volt until the purple tracer marker migrated to approximately 2 cm before the end of the gel. After electrophoresis, the gel was stained with ethidium bromide, and photographed under UV light as previously described [21].

### 2.7. Statistical Analysis

Data were presented as means ± SDs. Statistical analysis was done using one way analysis of variance (ANOVA Dunnett’s test) for multiple samples. Student’s paired test was used to analyze the difference between the controls and lead nitrate-treated cells. All *p*-values < 0.05 were considered to be significant. Graphs were made to illustrate the concentration-response relationship with respect to cell death, DNA damage, and apoptosis.

## 3. Results and Discussion

### 3.1. Lead Nitrate Inhibited Cell Growth

**Figure 1:**
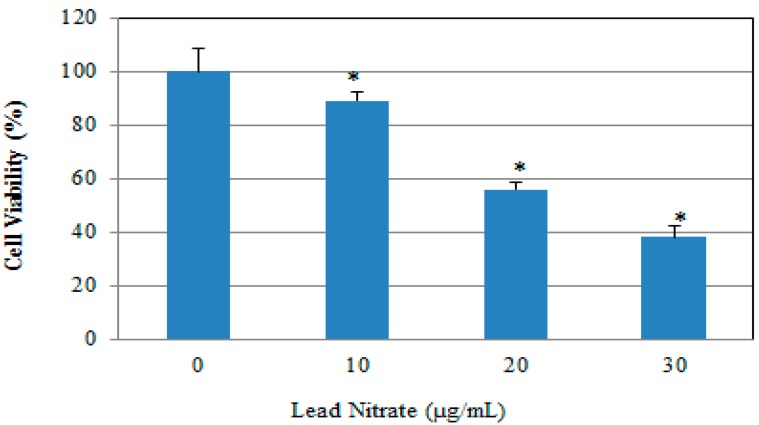
Cytotoxic effect of Pb(NO_3_)_2_ to HL-60 cells. HL-60 cells were cultured in the absence or presence of Pb(NO_3_)_2_ for 24 h. Cell viability was determined based on the propidium iodide assay. Each point represents a mean value of 3 experiments with 6 replicates per concentration. *p* < 0.05 *versus* compared with control group. * Significantly different (*p* < 0.05) from the control, according to the Dunnett’s test.

Cell viability of human leukemia (HL-60) cells in the absence and presence of lead was tested after 24 h exposure by propidium iodide assay based on cell density computed by the brightfield images using the cellometer vision. Figure 1 shows the results obtained after treatment of the HL-60 cells with three concentrations of lead nitrate including 10, 20 and 30 µg/mL lasting 24 h. A clear positive concentration-dependent relationship was observed in the percentage of cell death and increasing concentrations of lead nitrate (Figure 1). These results are agreement with those of previous investigations reporting a marked reduction in the viability of cancer cells following exposure to lead [22,23]. Recent study demonstrated that lead affects essentially every organ system in the body, including the hematopoietic, cardiovascular, renal and skeletal systems with the central nervous system being more sensitive to the effects of lead exposure [24]. The current reference lead concentration in blood set by Centre for Disease Control for identification and monitoring of children who are exposed to high level of lead is ≥5 µg/dL [25]. The blood lead level rises within hours of exposure and remains elevated for several weeks thereafter [26]. Due to lead’s short half-life time in the blood, blood lead tests cannot be used to diagnose or rule out evidence of exposure that occurred more than six weeks before testing. Studies show that even low-level exposures to lead impair cell-mediated immunity by upsetting the balance between Th1- and Th2- like T lymphocytes, which alters cytokine expression [27,28].

### 3.2. Lead Nitrate Induced Necrotic Cell Death

**Figure 2:**
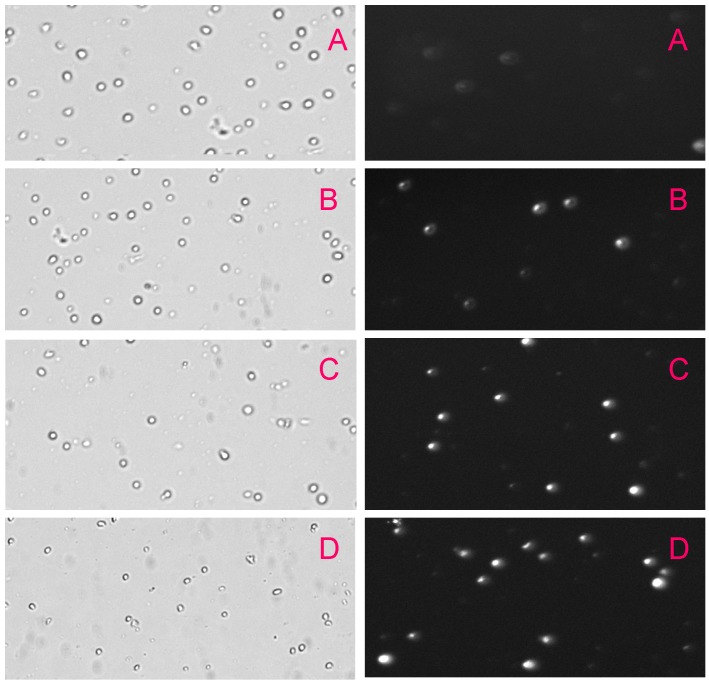
Bright field images (**left**) and fluorescent images (**right**) of HL-60 cells exposed to Pb(NO_3_)_2_ for 24 h. HL-60 cells were exposed to different concentrations of Pb(NO_3_)_2_. (**A**)—control; (**B**)—10 μg/mL Pb(NO_3_)_2_; (**C**)—20 μg/mL Pb(NO_3_)_2_; and (**D**)—30 μg/mL Pb(NO_3_)_2_. Images were taken using the Cellometer Vision (at 10× magnification).

We analyzed necrotic cell death in the absence and presence of Pb(NO_3_)_2_ after 24 h exposure by propidium iodide (PI) assay based on necrotic cells population computed by the fluorescent images using the Cellometer Vision. We found that lead nitrate induced necrotic cell death in a concentration-dependent manner (Figure 2). The number of cells stained with PI increased significantly in lead nitrate-treated cells compared with the control group. These results led us to conclude that lead nitrate induces necrosis in human leukemia (HL-60) cells. To the best of knowledge, we reported for the first time that lead nitrate is able to cause cell death through the necrosis pathway. As shown on Figure 2, brightfied images showed a gradual decrease in the cell viability of leukemic cells compared to the control while fluorescent images showed a gradual increase in the proportion of necrotic cell death with increasing concentrations of lead nitrate compared to the control. The fluorescent images showed strong morphological changes in lead-treated cells compared to the control group. Necrosis is a cell death process that is morphologically characterized by a gain in cell volume, swelling of organelles, plasma membrane rupture and subsequent loss of intracellular contents. This is in contrast to programmed cell death (apoptosis), although it was long thought that necrosis is an uncontrolled cell death that is characterized by progressive loss of cytoplasmic membrane integrity, rapid influx of Na^+^, Ca^2+^, and water, resulting in cytoplasmic swelling and nuclear pyknosis [29].

### 3.3. Lead Nitrate Induced Genotoxic Damage

**Figure 3:**
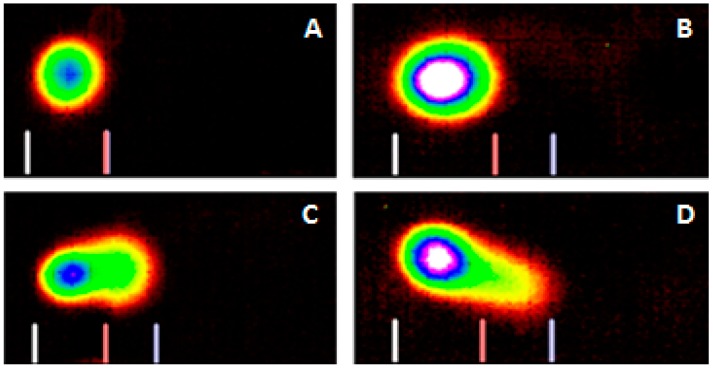
Representative SYBR Green Comet assay images of untreated ((**A**)—control) and Pb(NO_3_)_2_ treated HL-60 cells at 10 μg/mL (**B**); 20 μg/mL (**C**); and 30 μg/mL (**D**). High percentages (>45%) of selected images were observed in specific Pb(NO_3_)_2_ as indicated above.

**Figure 4:**
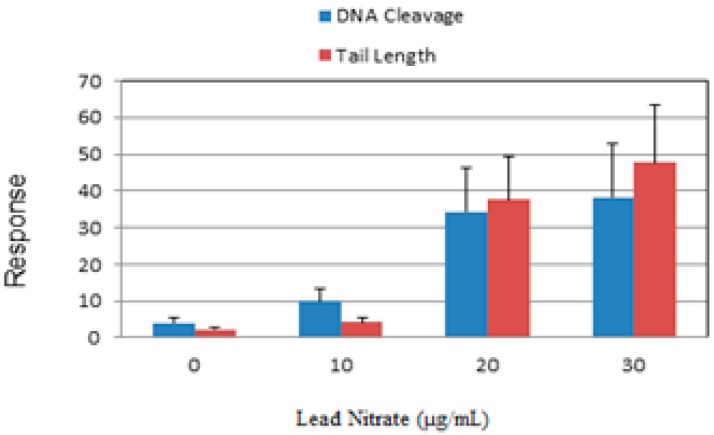
Comet assay of HL-60 cells showing the percentage of DNA cleavage (blue) and tail length (red), as a function of Pb(NO_3_)_2_ concentrations. Each point represents mean ± SD of 3 independent experiments.

The Comet assay is a highly sensitive technique to study DNA damage caused by metals [21,30]. In the present work, we used this technique to study lead nitrate-induced DNA damage in exposed HL-60 cells. Representative Comet assay images of control and lead nitrate-treated cells stained with SYBR Green are presented in Figure 3. As denoted in this figure, there is gradual increase in the mean values of comet tail length, tail moment, and percentages of DNA cleavage in HL-60 cells, with increasing concentrations of lead nitrate. The percentages of DNA cleavage and tail length are represented in Figure 4. Overall, the results generated from the comet assay indicated that lead nitrate is highly genotoxic to leukemia cells. Although it is generally accepted that lead is a possible genotoxic carcinogen in humans, it remains unclear how lead nitrate causes genetic damage in humans. Our Comet assay results indicate that lead nitrate exposure is able to induce DNA damage in human leukemia cells in concentration-dependent manner. This finding is in agreement with previous reports showing that lead causes destabilization of DNA, compaction and aggregation of chromatin, and impaired DNA and RNA synthesis [31] and Kupffer cell-mediated cell death in the liver [32]. Other reports indicated that lead causes point mutations in Chinese hamster ovary cells [33], abnormal base pairing [34], formation of micronuclei, chromosome aberration, and sister chromatid exchanges [35]. Studies by Roy and his group showed that lead acetate induced mutagenicity at a toxic dose at the *E. coli gpt* locus transfected to V79 cells [36]. They also reported that toxic doses of lead acetate and lead nitrate induced DNA breaks at the *E. coli gpt* locus transfected to V79 cells [36]. Another study by Wise and his collaborators found no evidence for direct genotoxic or DNA-damaging effects of lead except for lead chromate. They pointed out that the genotoxicity may be due to hexavalent chromate rather than lead [37].

### 3.4. Lead Nitrate Induced Cell Cycle Arrest

**Figure 5:**
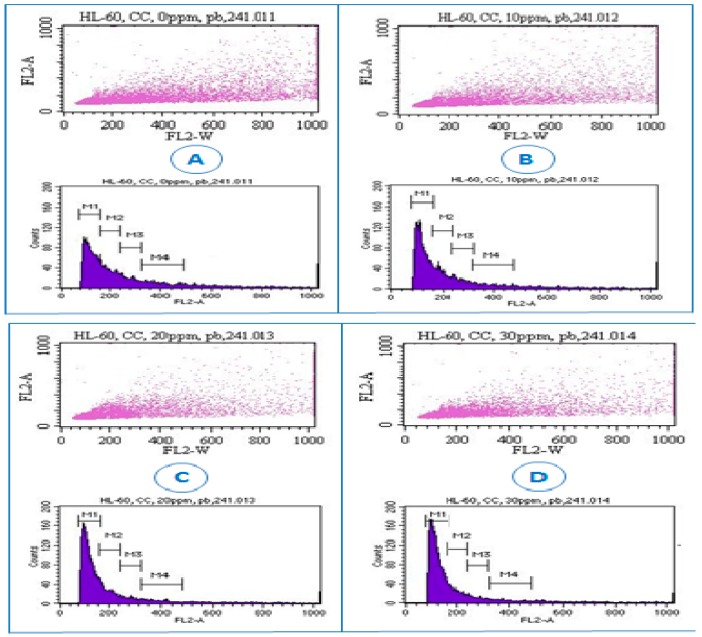
Representative dot plots and histograms showing cell cycle distribution in lead nitrate-treated HL-60 cells. Cell cycle distribution was determined by the propidium iodide staining method, and stained cells were analyzed by flow cytometry (FACS Calibar; Becton-Dickinson) using CellQuest software. A total of 10,000 cells were analyzed per sample. (**A**) control; (**B**) 10 μg/mL Pb(NO_3_)_2_; (**C**) 20 μg/mL Pb(NO_3_)_2_; (**D**) and 30 μg/mL Pb(NO_3_)_2_. Three experiments were performed, and 1 representative experiment is shown.

**Figure 6:**
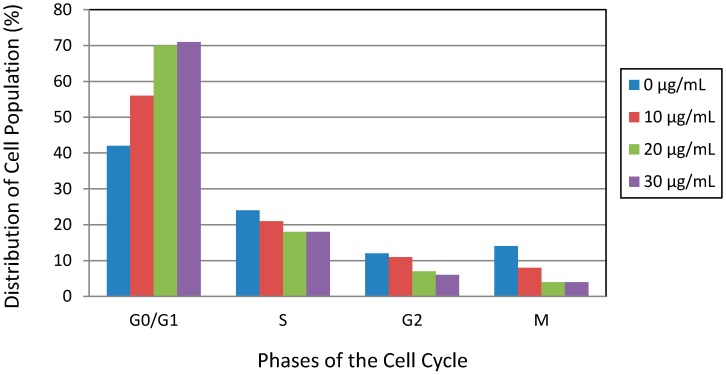
Lead nitrate-induced cell cycle arrest in human leukemia (HL-60) cells. Cells were cultured for 24 h with different concentrations of Pb(NO_3_)_2_. Cell cycle distribution was measured by the propidium iodide staining method. Representative results from at least 3 different experiments are shown.

To further confirm whether the growth inhibition of the cells induced by Pb(NO_3_)_2_ was related to cell cycle arrest, we analyzed cell cycle profile by flow cytometry assessment. The cell cycle is a highly regulated event that controls the growth and differentiation of cells [38,39]. Changes in cell cycle distribution might be associated with the apoptosis and differentiation of cells. We observed that Pb(NO_3_)_2_ exposure increased G0/G1 (M1 region) cell population from 42.2% in the control sample to 70.8% in treated sample at 30 µg/mL (Figure 5 and Figure 6). Meanwhile the S (M2 region) and G2/M (M3 and M4 regions) cell populations decreased with increasing concentrations of Pb(NO_3_)_2_ (Figure 5 and Figure 6). The increase of cell population at the G0/G1 and decrease of cell population at the S and G2/M phases suggested that Pb(NO_3_)_2_ caused cell cycle arrest at the G0/G1 checkpoint. To the best of our knowledge, no data was found in the literature regarding cell cycle arrest induced by lead in cell lines or animal models. Here, we report for the first time that Pb(NO_3_)_2_ is able to prominently induce cell cycle arrest at the G0/G1 checkpoint in HL-60 cell lines. In other studies with heavy metal toxicity, the antiproliferative action of arsenical compounds was linked to a G1 phase arrest in lymphoid neoplasms [40] and a G2-M phase arrest in NB4 cells [41] at lower doses.

### 3.5. Lead Nitrate Induced Apoptosis

**Figure 7:**
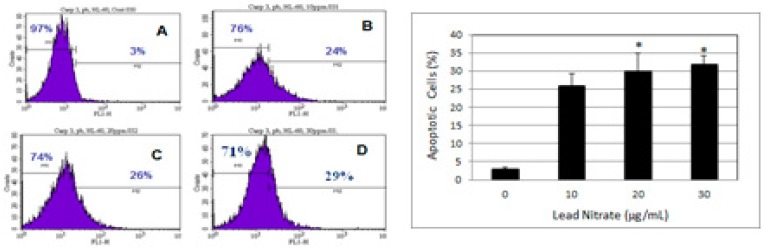
The histogram (**Left**) shows a comparison of the distribution of negative caspase-3 cells (M1) and positive caspase-3 cells (M2) after 24 h incubation in HL-60 cells. **A**—control; **B**—10 μg/mL Pb(NO_3_)_2_; **C**—20 μg/mL Pb(NO_3_)_2_; and **D**—30 μg/mL Pb(NO_3_)_2_. The bar graph (**Right**) shows the proportion of apoptotic cells in lead nitrate-treated cells compared to the control. Each point represents the mean value and the standard deviation of three experiments, showing similar results. * Significantly different from control (0 μg/mL), *p* < 0.05 [16].

**Figure 8:**
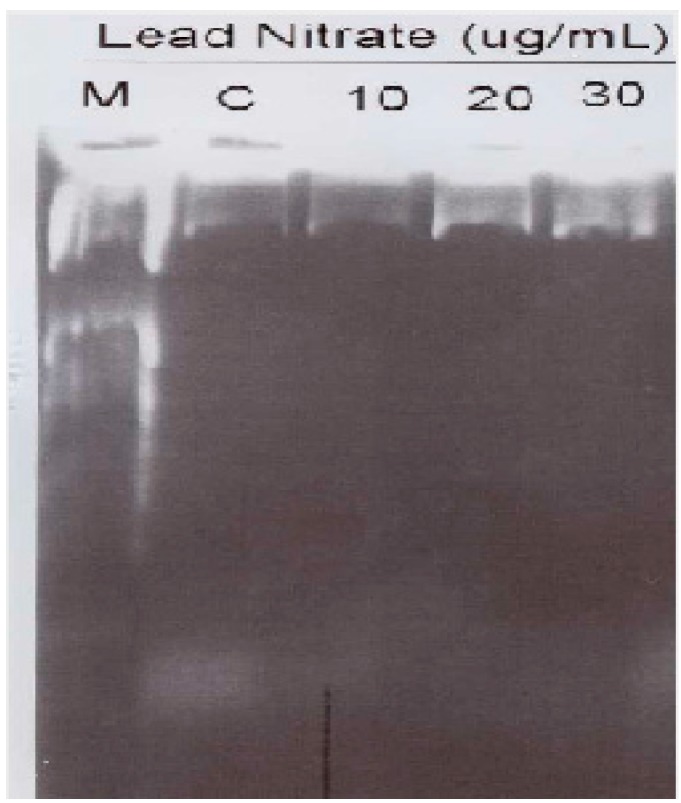
Lead nitrate induced DNA fragmentation in HL-60 cells. DNA marker (M) was electrophoresed as a base pair reference (lane 1). HL-60 cells were either untreated (Lane 2) or treated with 10, 20 and 30 μg/mL of Pb(NO_3_)_2_ (Lanes 3, 4, and 5) for 24 h. Chromosomal DNA was prepared using the apoptotic DNA ladder detection Kit according to the kit instructions. Twelve microliters of each sample was electrophoresed on a 1.2% agarose. DNA was stained with ethidium bromide after electrophoresis on a 1.2% agarose gel and then visualized under UV light.

Despite the fact that lead exposure has been the subject of intense research over many decades, the mechanisms responsible for its apoptotic effects are still poorly understood. A recent study in our laboratory demonstrated that lead exposure induced apoptosis in human leukemia cells at least in part through caspase-3 activation (Figure 7). As seen on Figure 7, the proportion of apoptotic cells (caspase-3 positive cells) increase with increasing concentration of Pb(NO_3_)_2_ in human leukemia (HL-60) cells. Caspase-3 plays an important role in the downstream of mitochondrial pathway, after dysfunction of mitochondria and the release of cytochrome c. It is well documented that caspase-3 activation occurs in lead induced apoptotic neurodegeneration [42] and apoptosis in HL-60 cells [16]. Caspase-3 is believed to be the final executor of apoptotic DNA damage [43]. In an attempt to further confirm that lead indeed induced apoptosis in human leukemia (HL-60) cells, we performed DNA laddering. Our result showed smear of nucleosomal DNA fragmentation in the nuclei isolated from HL-60 cells (Figure 8). Although we did not detect a direct presence of DNA fragments using the DNA laddering technique, we observed significant DNA damage in the nuclei of HL-60 cells by the comet assay technique (Figure 3). This observation leads us to suggest that nucleosomal DNA fragmentation may be involved in lead-induced apoptosis in human leukemia (HL-60) cells. DNA fragmentation is a biochemical hallmark of apoptosis. Induction of apoptosis has been recognized as a possible outcome of DNA damage for more than 35 years [44]. Studies indicated that apoptosis is associated with lead-induced toxicity in neuronal cells [45,46]. Recent report indicated that low-dose exposure to lead and Cadmium significantly cause hepatic and renal apoptosis and impair their function. Hepatic and renal apoptosis induced by low-dose exposure to cadmium and lead was found to be associated with mitochondrial injury and changes in levels of apoptogenic proteins including Bcl-2, Bax, and caspase-3 [47]. Using adult rat hepatic stem cells, Agarwal *et al*. reported that stimulation of caspase cascade and simultaneous extracellular signal-regulated kinase (ERK) dephosphorylation are the most significant operative pathways directly associated with apoptotic signals triggered by lead acetate [48].

## 4. Conclusions

In this study, the effect of Pb(NO_3_)_2_ as one of the hazardous heavy metals was studied at the cellular and molecular levels. From the results obtained in this work, we can conclude that Pb(NO_3_)_2_ inhibits the cell proliferation of HL-60 cell lines by not only inducing DNA damage and cell cycle arrest at the G_0_/G_1_ checkpoint but also triggering the apoptosis through caspase-3 activation and nucleosomal DNA fragmentation accompanied by secondary necrosis. As demonstrated in the present study, Pb(NO_3_)_2_ exposure has a strong cytotoxic, genotoxic, and apoptotic potential effects on human leukemia (HL-60) cells. According to the present finding, its mechanism of action includes growth inhibition of leukemia cells, induction of DNA damage, cell cycle arrest at the G_0_/G_1_ checkpoint, and apoptosis accompanied by secondary necrotic cell death. Hence, the present study provides a new insight into the mechanisms of Pb(NO_3_)_2_ induced toxicity and its associated adverse health effects.
